# Independently validated sex-specific nomograms for predicting survival in patients with newly diagnosed glioblastoma: NRG Oncology RTOG 0525 and 0825

**DOI:** 10.1007/s11060-021-03886-5

**Published:** 2021-11-10

**Authors:** Nirav Patil, Eashwar Somasundaram, Kristin A. Waite, Justin D. Lathia, Mitchell Machtay, Mark R. Gilbert, James R. Connor, Joshua B. Rubin, Michael E. Berens, Robin A. Buerki, Serah Choi, Andrew E. Sloan, Marta Penas-Prado, Lynn S. Ashby, Deborah T. Blumenthal, Maria Werner-Wasik, Grant K. Hunter, John C. Flickinger, Merideth M. Wendland, Valerie Panet-Raymond, H. Ian Robins, Stephanie L. Pugh, Minesh P. Mehta, Jill S. Barnholtz-Sloan

**Affiliations:** 1 University Hospitals, Cleveland, OH USA; 2 Case Western Reserve University School of Medicine, Cleveland, OH USA; 3 Division of Cancer Epidemiology and Genetics (DCEG), Trans-Divisional Research Program (TDRP) National Cancer Institute (NCI) National Institutes of Health (NIH), Shady Grove, MD USA; 4 Cleveland Clinic Foundation, Cleveland, OH USA; 5 Case Comprehensive Cancer Center, Cleveland, OH USA; 6 Penn State Milton S Hershey Medical Center, Hershey, PA USA; 7 National Cancer Institute, Neuro-Oncology Branch, Bethesda, MD USA; 8 Washington University of St Louis, St. Louis, MO USA; 9 TGen, Translational Genomics Research Institute, an Affiliate of City of Hope, Phoenix, AZ USA; 10 National Cancer Institute, Neuro-Oncology Branch; Accruals for University of Texas-MD Anderson Cancer Center (Houston, TX, USA), Bethesda, MD USA; 11 Barrow Neurology Clinics Accruals for Arizona Oncology Services Foundation, Phoenix, AZ USA; 12 Tel-Aviv Medical Center, Tel-Aviv University, Tel Aviv-Yafo, Israel; 13 Thomas Jefferson University Hospital, Philadelphia, PA USA; 14 Intermountain Medical Center, Salt Lake City, UT USA; 15 UPMC-Shadyside Hospital, Pittsburgh, PA USA; 16 USON- Willamette Valley Cancer Center, Eugene, OR USA; 17 McGill University Health Center, Montreal, QC Canada; 18 University of Wisconsin School of Medicine and Public Health, Madison, WI USA; 19 NRG Oncology Statistics and Data Management Center, American College of Radiology, Philadelphia, PA USA; 20 Miami Cancer Institute, Miami, FL USA; 21 Center for Biomedical Informatics and Information Technology (CBITT), National Cancer Institute (NCI)/National Institutes of Health (NIH), Shady Grove, MD USA

**Keywords:** Glioblastoma, Nomogram, Survival, Sex differences

## Abstract

**Background/purpose:**

Glioblastoma (GBM) is the most common primary malignant brain tumor. Sex has been shown to be an important prognostic factor for GBM. The purpose of this study was to develop and independently validate sex-specific nomograms for estimation of individualized GBM survival probabilities using data from 2 independent NRG Oncology clinical trials.

**Methods:**

This analysis included information on 752 (NRG/RTOG 0525) and 599 (NRG/RTOG 0825) patients with newly diagnosed GBM. The Cox proportional hazard models by sex were developed using NRG/RTOG 0525 and significant variables were identified using a backward selection procedure. The final selected models by sex were then independently validated using NRG/RTOG 0825.

**Results:**

Final nomograms were built by sex. Age at diagnosis, KPS, MGMT promoter methylation and location of tumor were common significant predictors of survival for both sexes. For both sexes, tumors in the frontal lobes had significantly better survival than tumors of multiple sites. Extent of resection, and use of corticosteroids were significant predictors of survival for males.

**Conclusions:**

A sex specific nomogram that assesses individualized survival probabilities (6-, 12- and 24-months) for patients with GBM could be more useful than estimation of overall survival as there are factors that differ between males and females. A user friendly online application can be found here—https://npatilshinyappcalculator.shinyapps.io/SexDifferencesInGBM/.

**Supplementary Information:**

The online version contains supplementary material available at 10.1007/s11060-021-03886-5.

## Introduction

Glioblastoma (GBM) represents 48.3% of all malignant primary brain tumors [1]. Despite advances in both treatment and biological understanding, prognosis remains poor. Other than the modest benefit demonstrated by the addition of temozolomide to radiotherapy, and TTField therapy to chemoradiotherapy, modern-day regimens have not significantly improved overall survival in the past 40 years [2–5]. According to an National Cancer Database study, long-term survivorship (over three years) in those with GBM is only ~ 9% [6].

While extent of resection, age at diagnosis, Karnofsky performance status (KPS), O-6-Methylguanine-DNA Methyltransferase (MGMT*)* promoter methylation status and presence of an *IDH1 or IDH2* mutation are well-validated prognostic factors, [7–9] more recently sex has been shown to be an important prognostic factor for GBM with better survival outcomes observed in females [6, 10]. Males have a higher incidence of GBM compared to females [1]. Transcriptome analysis has suggested the existence of sex-specific molecular subtypes for GBM indicating that the biological differences in disease likely extend beyond basic hormonal differences [11].

Currently, two nomograms have been developed for predicting 6-, 12-, and 24- month survival in GBM patients generally and in isocitrate dehydrogenase (IDH) wildtype GBM patients specifically [12, 13]. These nomograms use various demographic and biological factors as survival predictor variables including patient sex. We hypothesize that a sex-specific analysis may result in a more accurate survival prediction nomogram as sex was found be a significant predictor of survival in that analysis. The purpose of this study was to develop and independently validate sex-specific nomograms for estimation of individualized survival probabilities for GBM patients. We utilized data from 2 independent, recent, and non-overlapping NRG Oncology (formerly RTOG) clinical trials, NRG/RTOG 0525 and NRG/RTOG 0825 [14, 15].

## Methods

### Study population

Exempt approval was obtained from the University Hospitals Institutional Review Board (IRB) for all analyses presented. De-identified data were provided by NRG Oncology for the clinical trials NRG/RTOG 0525 and NRG/RTOG 0825 for which a written informed consent was obtained for each study subject under IRB approved protocols for each participating NRG study site [14, 15]. NRG/RTOG 0525 enrolled patients from January 2006 through June 2008; NRG/RTOG 0825 from April 2009 through May 2011. The two trials included information on 831 and 620 randomized patients with newly-diagnosed GBM, respectively. For each patient, the following variables were obtained: survival/follow-up time in months, survival status (dead or alive), progression-free survival time in months, progression-free survival status (no progression or progressed/dead), age at diagnosis (continuous), race (white, black, or other), sex (male or female), KPS (70, 80, 90, or 100), extent of resection (total/gross, subtotal, or other), MGMT promoter methylation status (promoter unmethylated or methylated), total number (0, 1, or ≥ 2) of comorbidities (heart problems, lung problems, high blood pressure, bleeding problems, circulation problems, diabetes, kidney/urine problems, stroke, thyroid problems, seizure, psychological problems), location of tumor within brain (frontal, temporal, parietal, occipital or multiple), laterality (right, left or bilateral) and use of corticosteroids (had to have received a stable or decreasing dose for the 5 days before study registration (yes/no)). Other category of extent of resection included unknown, biopsy, debulking, craniotomy etc. Overall, 88 patients with unknown MGMT promoter methylation status and 6 with unknown laterality were excluded from this analysis.

### Statistical analysis

Descriptive statistics were used to assess any differences in patient characteristics and prognostic factors by sex using t-tests for continuous variables and chi-square tests for categorical variables. Non-parametric equivalents were used as appropriate. The analyses were performed using NRG/RTOG 0525 as the training dataset and NRG/RTOG 0825 as the validation dataset. Both overall survival (OS) and progression-free survival (PFS) were examined for the trial dataset using the Kaplan–Meier method and were compared by sex using the log-rank test. Upon examination of the Shoenfeld residuals by sex, the proportional hazards assumption for all analyses by sex was not violated.

In the initial phase of nomogram development to select prognostic factors, we fit a multivariable Cox proportional hazards model by sex for both OS and PFS to the training set (0525). Cox models were found to be superior for survival prediction on these datasets in a previous publication [12], and a multivariable Cox model with sex as a variable using these datasets was reported in a previous publication [12]. In the first step, a model was fit by including every candidate survival predictor variable; in each subsequent step, the model with the smallest Akaike information criterion (AIC) score was chosen after removing one variable at a time (backward selection). And the model was refit with the remaining variables. This process was repeated until to the point where removing any variable would increase the AIC score. Criterion-based methods such as AIC are preferred as they involve a wider search and compare models in a preferable manner[16, 17]. The proportional hazards and linearity assumptions were examined using Schoenfeld and Martingale residuals. None of the variables included in the final model appear to violate these assumptions. We used the candidate variables retained by each sex specific Cox model on the training set (NRG/RTOG 0525) as the predictors of survival to independently validate (NRG/RTOG 0825) and build nomograms for OS and PFS. The final selected models were trained using the data from NRG/RTOG 0525 and were independently validated using the data from NRG/RTOG 0825.

Calibration of the final models by sex for both OS and PFS for both training and validation dataset was visually evaluated by assigning all patients into quintiles of the nomogram-predicted survival probabilities and plotting the mean nomogram predicted survival probability against the Kaplan–Meier estimated survival for each quintile. A user-friendly online application to obtain individualized predicted survival probabilities by sex was developed and can be found here—https://npatilshinyappcalculator.shinyapps.io/SexDifferencesInGBM/. All analysis were performed using R v3.6.0 (http://www.r-project.org/) and the online application was developed using R Shiny application.

## Results

### Patient characteristics

In both trials, treatment either did not affect primary outcomes (OS and PFS) or the outcomes did not reach the prespecified improvement target; therefore, the data from both of the studies were used in this analysis (1,359 patients in total across both trials). The comparison of patient characteristics between the trials is shown in Supplemental Table 1. Table 1 shows the patient characteristics by sex by trial. The proportion of males and females was similar in both trials (57.7% vs 60.3% males and 42.3% vs 39.7% females for NRG/RTOG 0525 and NRG/RTOG 0825, respectively). Males tended to have higher KPS scores, poorer OS, poorer PFS, and more cardiac co-morbidities. Tumor location and laterality did not significantly differ by sex. Extent of resection (EOR) also did not differ significantly by sex. The majority of patients included in this analysis had no comorbidities (45.9%) and there was no significant difference in total number of comorbidities by sex (Table 1).

**Table 1:** Patient characteristics by NRG Oncology Trial and sex

		NRG/RTOG 0525 (Training dataset)			NRG/RTOG 0825 (Validation dataset)		
	Level	Male (n = 434)	Female (n = 318)	P-value	Male (n = 361)	Female (n = 238)	P-value
Age at diagnosis	Mean (SD)	55.40 (12.13)	56.29 (11.60)	0.313^a^	57.89 (11.01)	57.32 (10.98)	0.532^a^
	Median (interquartile range)	57.00 [48.00, 64.00]	58.00 [50.00, 64.00]	0.242^b^	58.00 [52.00, 66.00]	58.00 [51.00, 64.00]	0.369^b^
Race, n (%)	Black	7 (1.6)	6 (1.9)	0.292^c^	3 (0.8)	7 (2.9)	0.091^c^
	Other/Unknown	98 (22.6)	57 (17.9)		9 (2.5)	9 (3.8)	
	White	329 (75.8)	255 (80.2)		349 (96.7)	222 (93.3)	
Karnofsky Performance Status at registration, n (%)	≤ 70	44 (10.1)	66 (20.8)	< 0.001^c^	38 (10.5)	38 (16.0)	0.238^c^
	80	98 (22.6)	48 (15.1)		96 (26.6)	63 (26.5)	
	90	176 (40.6)	141 (44.3)		161 (44.6)	94 (39.5)	
	100	116 (26.7)	63 (19.8)		66 (18.3)	43 (18.1)	
Extent of Resection, n (%)	Total or Gross total Resection	243 (56.0)	167 (52.5)	0.41^c^	212 (58.7)	153 (64.3)	0.249^c^
	Partial or Subtotal	176 (40.6)	143 (45.0)		137 (38.0)	81 (34.0)	
	Other	15 (3.5)	8 (2.5)		12 (3.3)	4 (1.7)	
Neurologic function, n (%)	No symptoms	168 (38.7)	94 (29.6)	0.053^c^	136 (37.7)	73 (30.7)	0.015^c^
	Minor symptoms	190 (43.8)	152 (47.8)		168 (46.5)	104 (43.7)	
	Moderate symptoms	33 (7.6)	29 (9.1)		20 (5.5)	16 (6.7)	
	Severe	43 (9.9)	43 (13.5)		37 (10.2)	45 (18.9)	
MGMT methylation status, n (%)	Methylated	120 (27.6)	119 (37.4)	0.006^c^	100 (27.7)	73 (30.7)	0.488^c^
	Unmethylated	314 (72.4)	199 (62.6)		261 (72.3)	165 (69.3)	
Overall survival status, n (%)	Alive	86 (19.8)	76 (23.9)	0.209^c^	109 (30.2)	95 (39.9)	0.018^c^
	Dead	348 (80.2)	242 (76.1)		252 (69.8)	143 (60.1)	
Overall Survival Time (months)*	Median (95% CI)	13.8 [12.4, 14.9]	17.9 [16.4, 20.1]	0.003^d^	15.7 [14.5, 16.6]	16.9 [15.2, 19.8]	0.03^d^
Progression-free survival status, n (%)	Alive without Pregression	38 (8.8)	33 (10.4)	0.532^c^	55 (15.2)	52 (21.8)	0.05^c^
	Progressed or death due to any cause	396 (91.2)	285 (89.6)		306 (84.8)	186 (78.2)	
Progression-free survival time (months)*	Median (95% CI)	5.8 [5.4, 6.4]	6.4 [5.8, 8.3]	0.06^d^	8.9 [7.8, 9.9]	10.3 [8.7, 12.3]	0.03^d^
Use of Steroids	Yes	359 (82.7)	253 (79.6)	0.315^c^	261 (72.3)	176 (73.9)	0.726^c^
Comorbidities							
Heart problems	Yes	44 (10.1)	14 (4.4)	0.006^c^	47 (13.0)	17 (7.1)	0.032^c^
Lung problems	Yes	12 (2.8)	16 (5.0)	0.154^c^	16 (4.4)	15 (6.3)	0.411^c^
High blood pressure	Yes	104 (24.0)	75 (23.6)	0.973^c^	138 (38.2)	80 (33.6)	0.288^c^
Bleeding problems	Yes	2 (0.5)	6 (1.9)	0.128^c^	6 (1.7)	2 (0.8)	0.622^c^
Circulation problems	Yes	8 (1.8)	5 (1.6)	0.999^c^	8 (2.2)	4 (1.7)	0.873^c^
Diabetes	Yes	35 (8.1)	22 (6.9)	0.655^c^	46 (12.7)	15 (6.3)	0.016^c^
Kidney/urine problems	Yes	12 (2.8)	4 (1.3)	0.246^c^	23 (6.4)	14 (5.9)	0.944^c^
Stroke	Yes	4 (0.9)	5 (1.6)	0.637^c^	13 (3.6)	3 (1.3)	0.139^c^
Thyroid problems	Yes	8 (1.8)	45 (14.2)	< 0.001^c^	21 (5.8)	46 (19.3)	< 0.001^c^
Seizure	Yes	59 (13.6)	52 (16.4)	0.343^c^	52 (14.4)	32 (13.4)	0.833^c^
Psychological problems	Yes	16 (3.7)	7 (2.2)	0.340^c^	12 (3.3)	7 (2.9)	0.981^c^
Total number of Comorbidities	None	239 (55.1)	159 (50.0)	0.388^c^	128 (35.5)	95 (39.9)	0.525^c^
	1	112 (25.8)	91 (28.6)		132 (36.6)	79 (33.2)	
	≥ 2	83 (19.1)	68 (21.4)		101 (28.0)	64 (26.9)	
Location of Tumor In Brain	Frontal Lobe	115 (26.5)	102 (32.1)	0.013^c^	83 (23.0)	61 (25.6)	0.753^c^
	Occipital Lobe	17 (3.9)	16 (5.0)		7 (1.9)	6 (2.5)	
	Parietal Lobe	62 (14.3)	58 (18.2)		49 (13.6)	25 (10.5)	
	Temporal Lobe	148 (34.1)	72 (22.6)		93 (25.8)	58 (24.4)	
	Multiple	92 (21.2)	70 (22.0)		129 (35.7)	88 (37.0)	
Laterality	Right	237 (54.6)	181 (56.9)	0.780^c^	198 (54.8)	128 (53.8)	0.936^c^
	Left	190 (43.8)	133 (41.8)		158 (43.8)	106 (44.5)	
	Bilateral	7 (1.6)	4 (1.3)		5 (1.4)	4 (1.7)	

Overall Survival Time—Time since randomization to death/last follow-up

Progression-free survival time—Time since randomization to progression or date of death, or date of last-follow-up if alive without progression

88 patients with unknown MGMT status, 6 with unknown laterality, 2 with missing survival months and 8 with unknown location of tumor were excluded

Very small number of patients had Liver disease (n = 12), HIV (n = 2) and infections (n = 9)

*CI* Confidence Interval

^*^Kaplan Meier survival times

^a^Independent t test

^b^Mann-Whitney test

^c^Chi-square test

^d^Log rank test

### Survival by the Kaplan–Meier method

**Fig. 1:**
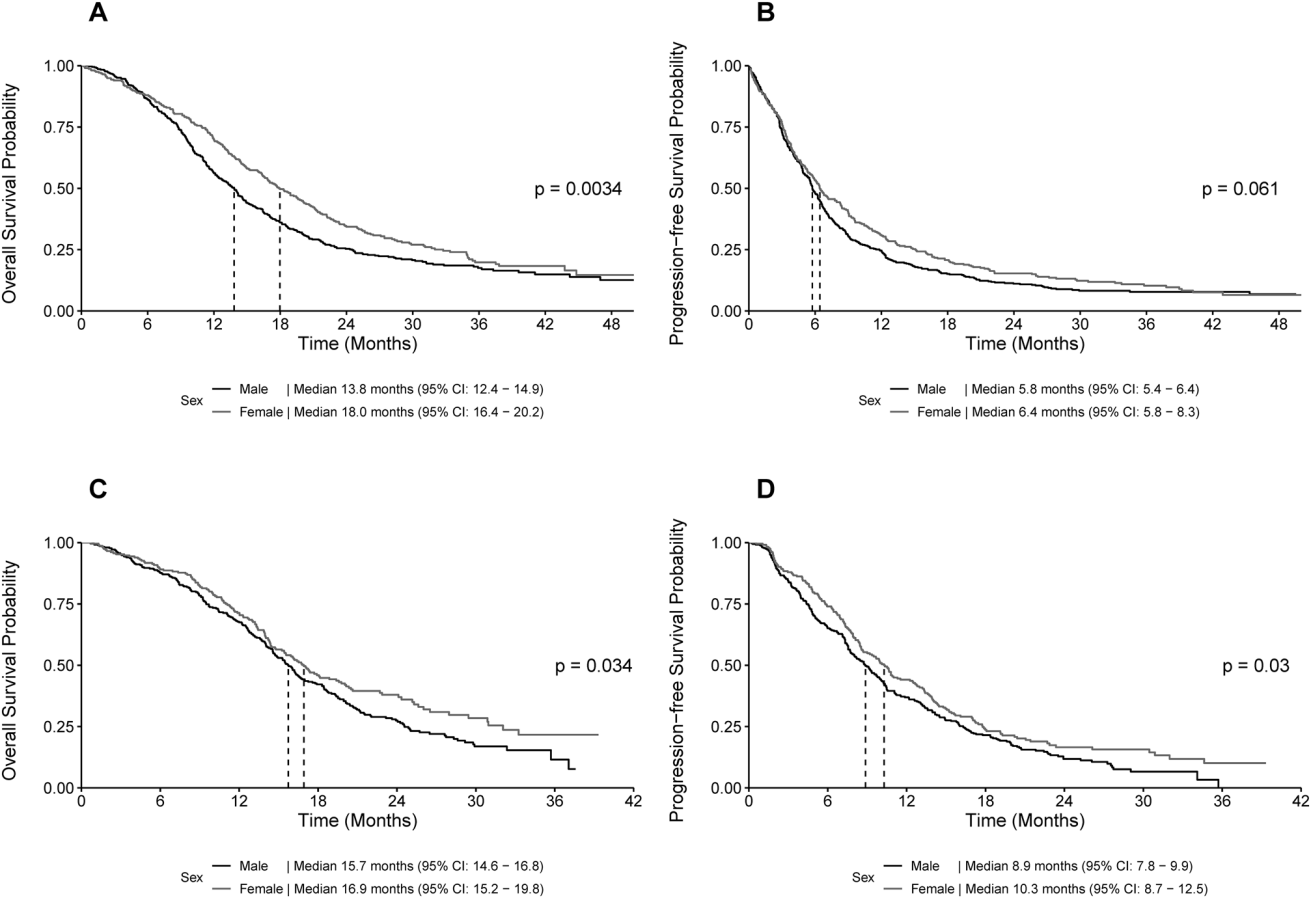
Kaplan–Meier Survival Results by Sex for Overall and Progression-Free Survival Using Training (NRG/RTOG 0525) (**A** and **B**) and Validation (NRG/RTOG 0825) (**C** and **D**) datasets

Kaplan–Meier curves were generated for OS and PFS for both NRG/RTOG 0525, the training dataset (Fig. 1 Panels A and B) and NRG/RTOG 0825 (Fig. 1 Panels C and D), the validation dataset. In the training dataset, females had a median survival of 17.9 months (16.4–20.1), which differed significantly from male OS of 13.8 months (12.4–14.9) (log rank p = 0.003). Males also had poorer PFS of 5.8 months (5.4–6.4) compared to female PFS of 6.4 months (5.8–8.3) but this was not significant (log rank p = 0.06). In the validation dataset, females had a significantly greater median survival of 16.9 months (15.2–19.8) compared to male median survival of 15.7 months (14.5–16.6, log rank p = 0.03). The PFS was significantly different between females (10.3 months, 8.7–12.3) and males (8.9 months, 7.8–9.9, log rank p = 0.03). These differences in the median survival were unadjusted estimates.

### Sex differences in survival

The overall Cox model by sex with the variables selected in the final model is shown in Table 2 for OS and Supplemental Table 4 for PFS. Based on the AIC criteria, age at diagnosis, KPS, MGMT status and location of tumor were common significant predictors of survival for both sexes. Extent of resection and use of corticosteroids were significant predictors of OS for males. However, for both sexes, tumors in frontal lobe had significantly better survival than tumors involving multiple sites. There was no difference in survival between other sites and tumors of multiple sites. Age, and MGMT status were also significant predictors for PFS for both sexes.

**Table 2:** Final Multivariable Cox Proportional Hazards Results for Overall Survival by Sex using the Training Dataset (NRG/RTOG 0525)

	Male Overall Survival						Female Overall Survival				
	N	Died	HR		95% CI	p-value	N	Died	HR^1^	95% CI^1^	p-value
Age at Diagnosis	434	348	1.02		1.01, 1.03	< 0.001	318	242	1.03	1.02, 1.05	< 0.001
Karnofsky Performance Status at registration											
< = 70	44	40 (90.9%)	—		—		66	55 (83.3%)	—	—	
80	98	86 (87.8%)	0.88		0.59, 1.30	0.505	48	40 (83.3%)	0.68	0.45, 1.03	0.067
90	176	139 (79.0%)	0.61		0.42, 0.88	0.008	141	106 (75.2%)	0.53	0.38, 0.75	< 0.001
100	116	83 (71.6%)	0.50		0.34, 0.74	< 0.001	63	41 (65.1%)	0.51	0.34, 0.76	0.001
Extent of Resection											
GTR	243	192 (79.0%)	—		—		167	119 (71.3%)	—	—	
STR	176	141 (80.1%)	1.23		0.98, 1.54	0.070	143	117 (81.8%)	—	—	—
Other	15	15 (100.0%)	2.01		1.17, 3.46	0.012	8	6 (75.0%)	—	—	—
MGMT methylation status											
Unmethylated	314	267 (85.0%)	—		—		199	164 (82.4%)	—	—	
Methylated	120	81 (67.5%)	0.54		0.42, 0.70	< 0.001	119	78 (65.5%)	0.51	0.38, 0.67	< 0.001
Use of Corticosteroids											
No	75	55 (73.3%)	—		—		65	46 (70.8%)	—	—	—
Yes	359	293 (81.6%)	1.35		1.01, 1.81	0.046	235	196 (77.5%)	—	—	—
Location of Tumor In Brain											
Multiple Sites	92	80 (87.0%)	—		—		70	58 (82.9%)	—	—	
Frontal Lobe	115	79 (68.7%)	0.66		0.48, 0.91	0.010	102	69 (67.6%)	0.62	0.44, 0.89	0.009
Occipital Lobe	17	12 (70.6%)	0.73		0.39, 1.34	0.306	16	12 (75.0%)	0.87	0.46, 1.64	0.673
Parietal Lobe	62	53 (85.5%)	1.05		0.73, 1.49	0.805	58	48 (82.8%)	0.77	0.52, 1.14	0.191
Temporal Lobe	148	124 (83.8%	1.02		0.76, 1.36	0.912	72	55 (76.4%)	0.79	0.54, 1.15	0.223

Variables not included in the table were not included in the final model. Extent of Resection and Use of Corticosteroids were not included in the final model for females

*HR* Hazard Ratio, *CI* Confidence Interval

### Nomograms

**Fig. 2:**
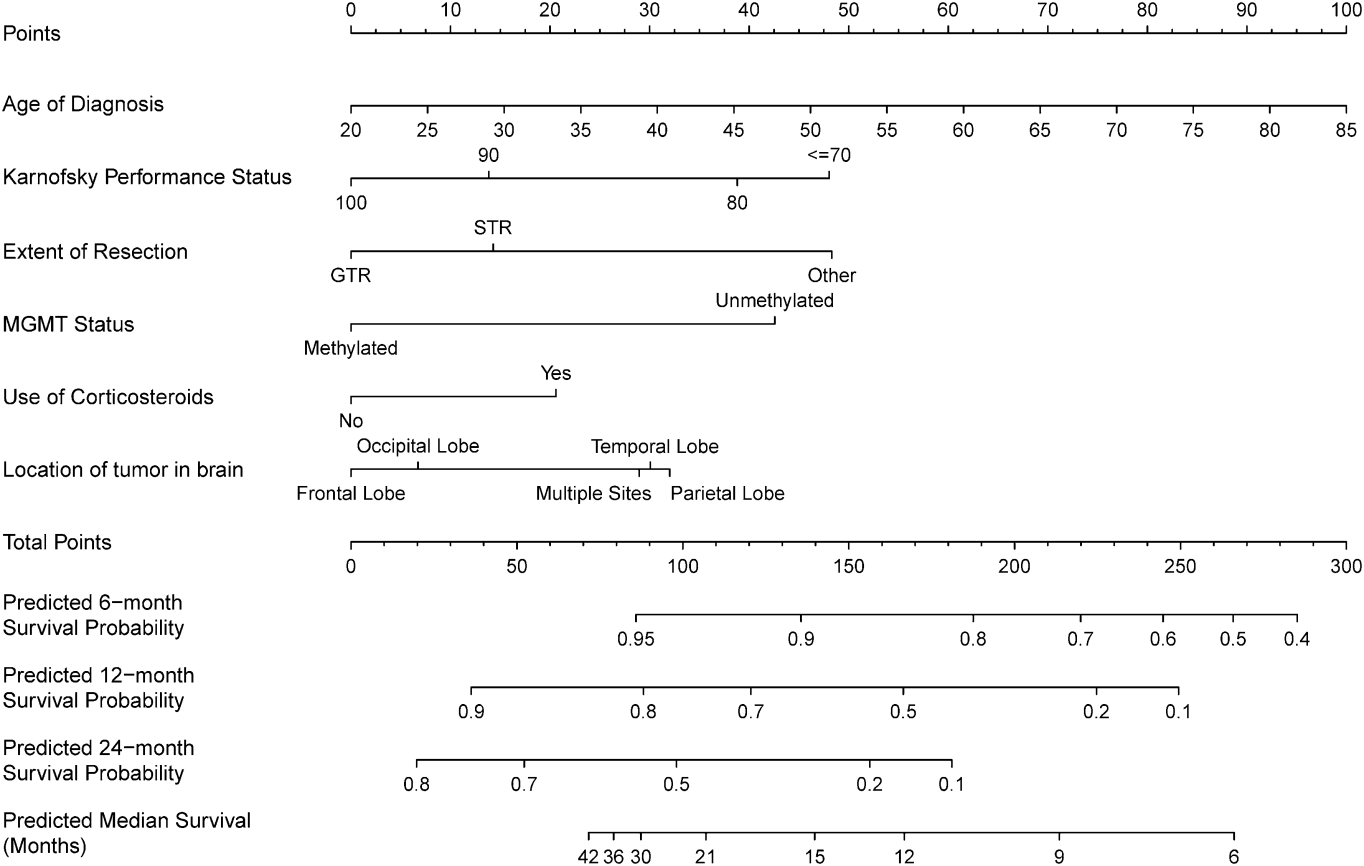
Final nomogram of Overall Survival for Males built on training data (NRG/RTOG 0525) and independently validated on NRG/RTOG 0825

**Fig. 3:**
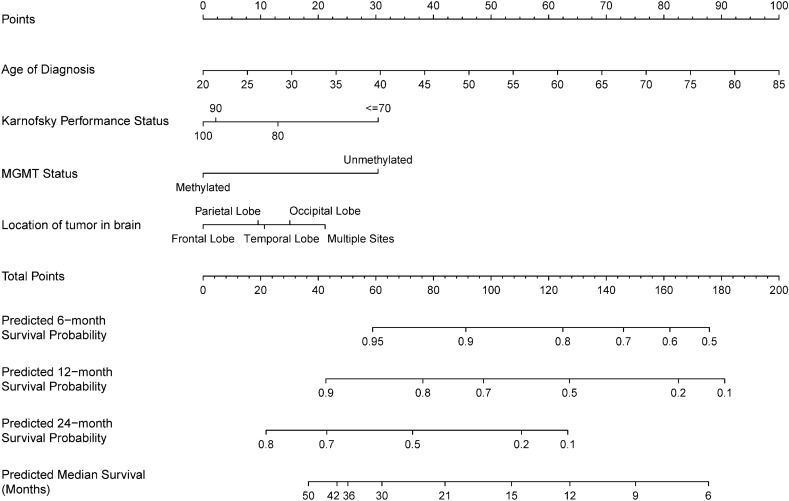
Final nomogram of Overall Survival for Females built on training data NRG/RTOG 0525 and independently validated on NRG/RTOG 0825

Calibration curves were drawn for both training (NRG/RTOG 0525) and validation (NRG/RTOG 0825) datasets for predicted 6-, 12-, and 24-month overall survival by sex (Supplemental Figs. 1 and 2). The curves show three lines, blue (observed survival rates), gray (ideal survival rates), and black (optimism/bias/ overfitting corrected survival rates). The 12-month and 24-month survival, observed and optimism corrected lines, are nearly identical showing near perfect calibration for OS. A sex-specific nomogram was developed for OS (Figs. 2 and 3). All nomograms were developed using NRG/RTOG 0525 as the training data and validated with NRG/RTOG 0825. The calibration curves for validation datasets were plotted using parameters from model using training dataset. The final multivariable model for validation dataset is shown in Supplemental Table 3. The calibration curves for PFS were not as accurate as those for OS (Supplemental Figs. 3 and 4). In addition, progression was determined by site investigator’s determination rather than centrally reviewed PFS standards, hence reducing the validity of this measure. For these reasons, we did not validate or construct nomograms for PFS.

## Discussion

In this study, we sought to develop and independently validate, sex-specific individual prognostic nomograms for patients with newly-diagnosed GBM. Our analysis includes a large group of GBM patients from 2 modern clinical trials. In the original NRG/RTOG 0525 and 0825 clinical trials, OS and PFS were not significantly different in treatment or control arms [14, 15]. This allowed us to train models on 0525 and externally validate using data from 0825 with no further adjustment for treatment arms. For OS in the male and female calibration curves, the ideal, bias-corrected, and observed curves tracked closely to each other for training and validation data. This suggests that the nomogram is resistant to possible batch effect and overfitting. In addition, the use of backward selection based on AIC to select only the most important variables prevents overfitting from using excess variables. In contrast, the calibration curves for PFS were not as strong, therefore we did not develop nomograms.

Interestingly, the factors that contribute to PFS and OS differ between males and females. Based on the final selected variables, age of diagnosis, KPS score, MGMT-promoter methylation status, extent of resection, use of corticosteroids, and location of the tumor in the brain are the significant predictors of OS for males. However, extent of resection was not a significant predictor of OS for females likely due to very low sample size for females with ‘Other’ resection (Table 1). For PFS, age at diagnosis*,* MGMT-promoter methylation status and extent of resection were significant survival predictors for males. In females, however, KPS score was significant and extent of resection was not a significant predictor of PFS. Similar to OS, the inconclusive p-values for some variables were likely due to very low sample size for both sexes.

While some of the variables for OS are the same for both males and females, the relative importance of these factors in terms of total points on the nomogram is different. The total point distribution for age of diagnosis, MGMT promoter methylation status and KPS are significantly higher for males compared to females indicating worse survival for males compared to females. This finding is similar to what has been reported earlier with these datasets, although these results were not stratified by sex [12]. However, there are some differences with respect to factors affecting survival by sex. Interestingly, the impact of extent of resection is different between males and females, albeit this could be due to lower sample size in females. Maximal extent of resection is currently equally indicated regardless of sex. It should be noted that extent of resection is a complex and somewhat subjective variable that incorporates abilities of the treating neurosurgeon, tumor size, tumor location as it related to proximity to eloquent cerebral cortex and other intracranial structures, dominant vs non-dominant laterality and the patient’s general medical risks. Moreover, extent of resection generally does not consider resected or residual non-contrast enhancing disease.

Location of the tumor in the brain also had different impact on OS and PFS between males and females. While tumors in the frontal lobe had significantly better survival probability compared to tumor involving multiple sites for both sexes, tumors at the other locations did not have any advantage over tumors in multiple sites. Further research is needed to validate this finding and to translate it to clinical relevance as we did not see similar association in the validation dataset. Additionally, the total number of comorbidities was not found to be significant for either sex possibly due to the fact that a large number of patients included in these trials did not have any comorbidity or only a small number of patients had each comorbidity (Table 1). We examined the univariate association of each of the comorbidity with OS by sex and found that none of the comorbidities were significant, except lung disease which was marginally significant (Supplemental Table 2). The impact of these comorbidities on the survival should be investigated in future trials with a larger sample size.

The primary limitations in our work include demographic differences between the two NRG clinical trials; and the population of GBM patients as a whole. While the patient demographics across both NRG trials are similar, race distribution, extent of resection patterns, and number of comorbidities varied between the studies. NRG/RTOG 0825, the validation set, had more white patients, greater gross total resection, and fewer patient comorbidities. All of these factors have been repeatedly shown to be prognostic for GBM survival [12, 6, 8]. However, in both the training (NRG/RTOG 0525) and validation (NRG/RTOG 0825) datasets, white patients were disproportionally more represented compared to distribution of GBM in the larger US population^7^. This may be the reason race was not found to be a significant factor. The patients in both trials may not be fully representative of the entire GBM population due to trial eligibility requirements. NRG/RTOG 0525 and 0825 had KPS cutoffs at 60 and 70 respectively and required adequate hematological, renal, and hepatic function [14, 15]. As such, the nomograms may not be predictive of survival in patients who have clinical characteristics different from the inclusion criteria of these clinical trials. The presence of an IDH mutation defines a separate entity from IDH-wildtype glioblastoma and is prognostic of survival outcomes. However, these studies predated routine testing of this biomarker and hence IDH mutation status was not available for the trials used in this study[9, 18]. Besides, IDH mutation only occurs in a small proportion of GBMs, hence these nomograms would be applicable for the majority of patients [19]. Finally, PFS in these older NRG/RTOG trials is based upon site investigator determination rather than central reviewers. Caution should be used when applying these nomograms to patients who are demographically or medically different from the population included in this analysis. Lastly, PFS should not be presumed to be a reliable endpoint, as the determination of progression was not by central review, and may have included instances of pseudoprogression.

The differences in the nomograms by sex shown here indicates that the prognosis of females and males may be different and that these nomograms are useful tools for estimating patient-level survival probabilities. To facilitate clinical use of this nomogram, free software for its implementation is provided (https://npatilshinyappcalculator.shinyapps.io/SexDifferencesInGBM/). This tool will be useful to health care providers in determining individualized survival probabilities by sex. Further research should be done to better characterize the exact biological mechanisms underlying sex differences in GBM.

## Supplementary Information

Below is the link to the electronic supplementary material.

Supplementary file1 (DOCX 296 kb)

## Data Availability

The datasets analyzed for this study are available by request from NRG.
