# Supplementary material for: Independently validated sex-specific nomograms for predicting survival in patients with newly diagnosed glioblastoma: NRG Oncology RTOG 0525 and 0825
Source: J Neurooncol. 2021 Nov 10;155(3):363–72. doi: 10.1007/s11060-021-03886-5 (PMC8651582; doi:10.1007/s11060-021-03886-5)

**Supplemental Table 1: Patient Characteristics by NRG Oncology Radiation Therapy Oncology Group (RTOG) Trial**

|  | **Level** | **Overall**  **N = 1,351** | **RTOG 0525**  **(Training dataset)**  **n = 752** | **RTOG 0825 (Validation Dataset)**  **n = 599** | **P-value** |
| --- | --- | --- | --- | --- | --- |
| **Age at diagnosis** | Mean (SD) | 56.6 (11.5) | 55.8 (11.9) | 57.7 (11.0) | 0.003 |
|  | Median (interquartile range) | 58.0 (50.0, 64.5) | 57.0 (48.0, 64.0) | 58.0 (51.0, 65.0) | 0.009 |
| **Race, n (%)** | Black | 23 (1.7%) | 13 (1.7%) | 10 (1.7%) | <0.001 |
|  | Other/Unknown | 173 (13%) | 155 (21%) | 18 (3.0%) |  |
|  | White | 1,155 (85%) | 584 (78%) | 571 (95%) |  |
| **Karnofsky Performance Status at registration, n (%)** | ≤ 70 | 186 (14%) | 110 (15%) | 76 (13%) | 0.004 |
|  | 80 | 305 (23%) | 146 (19%) | 159 (27%) |  |
|  | 90 | 572 (42%) | 317 (42%) | 255 (43%) |  |
|  | 100 | 288 (21%) | 179 (24%) | 109 (18%) |  |
| **Sex, n (%)** | Male | 795 (59%) | 434 (58%) | 361 (60%) | 0.343 |
|  | Female | 556 (41%) | 318 (42%) | 238 (40%) |  |
| **Extent of Resection, n (%)** | Total or Gross total Resection | 775 (57%) | 410 (55%) | 365 (61%) | 0.060 |
|  | Partial or Subtotal | 537 (40%) | 319 (42%) | 218 (36%) |  |
|  | Other | 39 (2.9%) | 23 (3.1%) | 16 (2.7%) |  |
| **Neurologic function, n (%)** | No symptoms | 471 (35%) | 262 (35%) | 209 (35%) | 0.301 |
|  | Minor symptoms | 614 (45%) | 342 (45%) | 272 (45%) |  |
|  | Moderate symptoms | 98 (7.3%) | 62 (8.2%) | 36 (6.0%) |  |
|  | Severe | 168 (12%) | 86 (11%) | 82 (14%) |  |
| **MGMT methylation status, n (%)** | Methylated | 412 (30%) | 239 (32%) | 173 (29%) | 0.250 |
|  | Unmethylated | 939 (70%) | 513 (68%) | 426 (71%) |  |
| **Overall survival status, n (%)** | Alive | 366 (27%) | 162 (22%) | 204 (34%) | <0.001 |
|  | Dead | 985 (73%) | 590 (78%) | 395 (66%) |  |
| **Overall Survival Time (months)** | Median (IQR) | 14.7 (9.1, 22.6) | 14.8 (9.1, 25.4) | 14.7 (9.0, 20.5) | 0.016 |
| **Progression-free survival status, n (%)** | Alive without Pregression | 178 (13%) | 71 (9%) | 107 (18%) | <0.001 |
|  | Progressed or death due to any cause | 1,173 (87%) | 681 (91%) | 492 (82%) |  |
| **Progression-free survival time (months)** | Median (IQR) | 7.2 (3.4, 14.5) | 6.0 (2.9, 12.4) | 8.7 (4.5, 15.9) | <0.001 |
| **Use of Steroids** | Yes | 1,049 (78%) | 612 (81%) | 437 (73%) | <0.001 |
| **Comorbidities** | | | | | |
| **Heart problems** | Yes | 122 (9.0%) | 58 (7.7%) | 64 (11%) | 0.058 |
| **Lung problems** | Yes | 59 (4.4%) | 28 (3.7%) | 31 (5.2%) | 0.195 |
| **High blood pressure** | Yes | 397 (29%) | 179 (24%) | 218 (36%) | <0.001 |
| **Bleeding problems** | Yes | 16 (1.2%) | 8 (1.1%) | 8 (1.3%) | 0.646 |
| **Circulation problems** | Yes | 25 (1.9%) | 13 (1.7%) | 12 (2.0%) | 0.710 |
| **Diabetes** | Yes | 118 (8.7%) | 57 (7.6%) | 61 (10%) | 0.092 |
| **Kidney/urine problems** | Yes | 53 (3.9%) | 16 (2.1%) | 37 (6.2%) | <0.001 |
| **Stroke** | Yes | 25 (1.9%) | 9 (1.2%) | 16 (2.7%) | 0.046 |
| **Thyroid problems** | Yes | 120 (8.9%) | 53 (7.0%) | 67 (11%) | 0.008 |
| **Seizure** | Yes | 195 (14%) | 111 (15%) | 84 (14%) | 0.702 |
| **Psychological problems** | Yes | 42 (3.1%) | 23 (3.1%) | 19 (3.2%) | 0.905 |
| **Total number of Comorbidities** | None | 621 (46%) | 398 (53%) | 223 (37%) | <0.001 |
|  | 1 | 414 (31%) | 203 (27%) | 211 (35%) |  |
|  | ≥ 2 | 316 (23%) | 151 (20%) | 165 (28%) |  |
| **Location of Tumor In Brain** | Frontal Lobe | 361 (27%) | 217 (29%) | 144 (24%) | <0.001 |
|  | Occipital Lobe | 46 (3.4%) | 33 (4.4%) | 13 (2.2%) |  |
|  | Parietal Lobe | 194 (14%) | 120 (16%) | 74 (12%) |  |
|  | Temporal Lobe | 371 (27%) | 220 (29%) | 151 (25%) |  |
|  | Multiple | 379 (28%) | 162 (22%) | 217 (36%) |  |
| **Laterality** | Right | 744 (55%) | 418 (56%) | 326 (54%) | 0.913 |
|  | Left | 587 (43%) | 323 (43%) | 264 (44%) |  |
|  | Bilateral | 20 (1.5%) | 11 (1.5%) | 9 (1.5%) |  |
| Overall Survival Time - Time since randomization to death/last follow-up  Progression-free survival time - Time since randomization to progression or date of death, or date of last-follow-up if alive without progression  a – Independent t test, b – Mann-Whitney test, c – Chi-square test  88 patients with unknown MGMT status, 6 with unknown laterality, 2 with missing survival months and 8 with unknown location of tumor were excluded  Very small number of patients had Liver disease (n = 12), HIV (n = 2) and infections (n = 9) | | | | | |

**Supplemental Table 2: Univariable Cox Proportional Hazards P-value of Individual Comorbidities with Survival by Sex using Training Dataset (0525)**

|  | **Male OS** | **Female OS** |
| --- | --- | --- |
| Heart problems | 0.368 | 0.276 |
| Lung problems | 0.046 | 0.047 |
| High blood pressure | 0.961 | 0.149 |
| Bleeding problems | 0.448 | 0.107 |
| Circulation problems | 0.943 | 0.376 |
| Diabetes | 0.334 | 0.196 |
| Kidney/urine problems | 0.802 | 0.943 |
| Stroke | 0.404 | 0.954 |
| Thyroid problems | 0.243 | 0.023 |
| Seizure | 0.103 | 0.559 |
| Psychological problems | 0.542 | 0.249 |

**Supplemental Figure 1. Male Overall Survival (A-F) Calibration curves for the nomogram training data (0525) at 6 months (A), 12 months (B), and 24 months (C) and for independent validation data (0825) at 6 months (D), 12 months (E), and 24 months (F).**


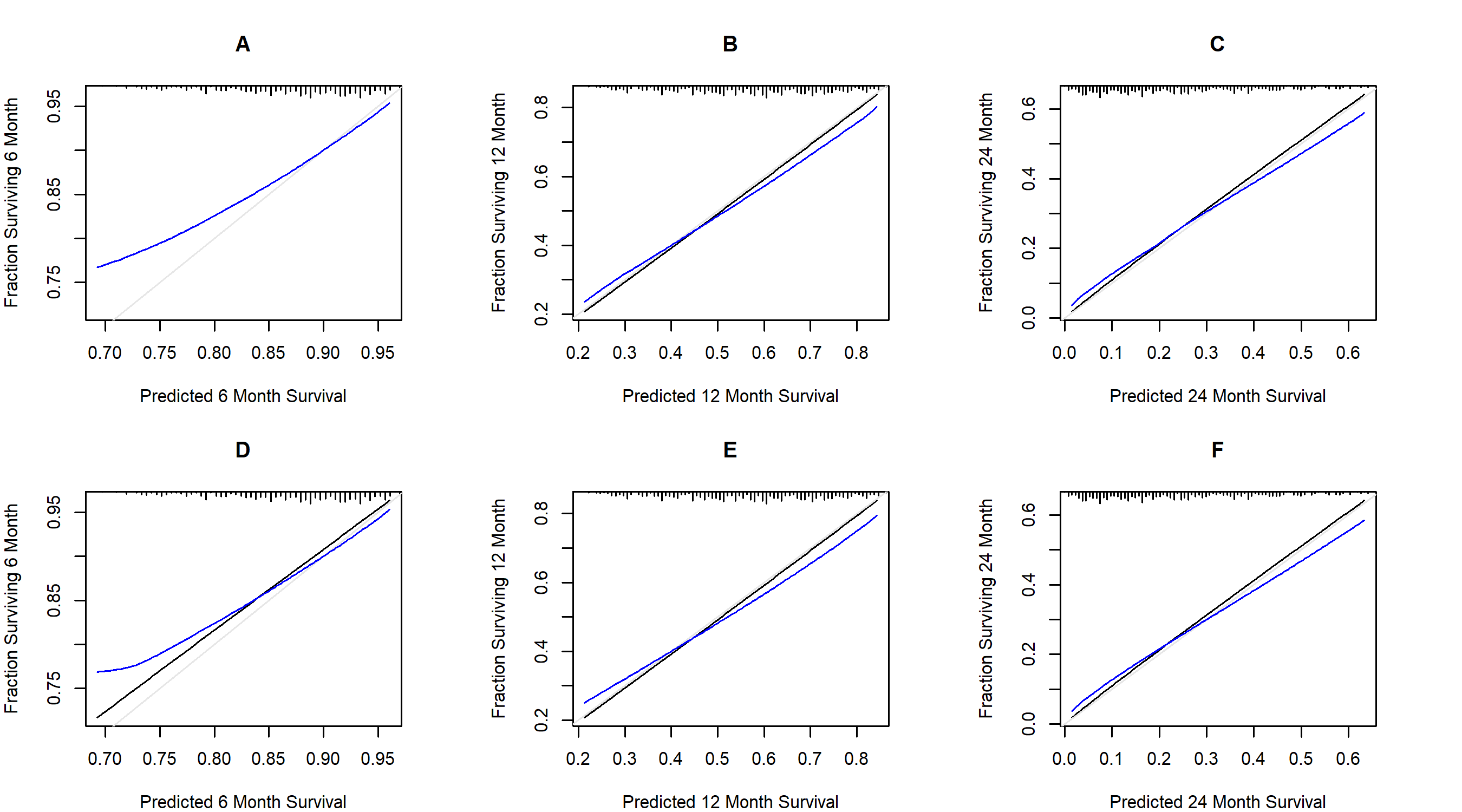


**Supplemental Figure 2. Female Overall Survival (A-F) Calibration curves for the nomogram training data (0525) at 6 months (A), 12 months (B), and 24 months (C) and for independent validation data (0825) at 6 months (D), 12 months (E), and 24 months (F).**


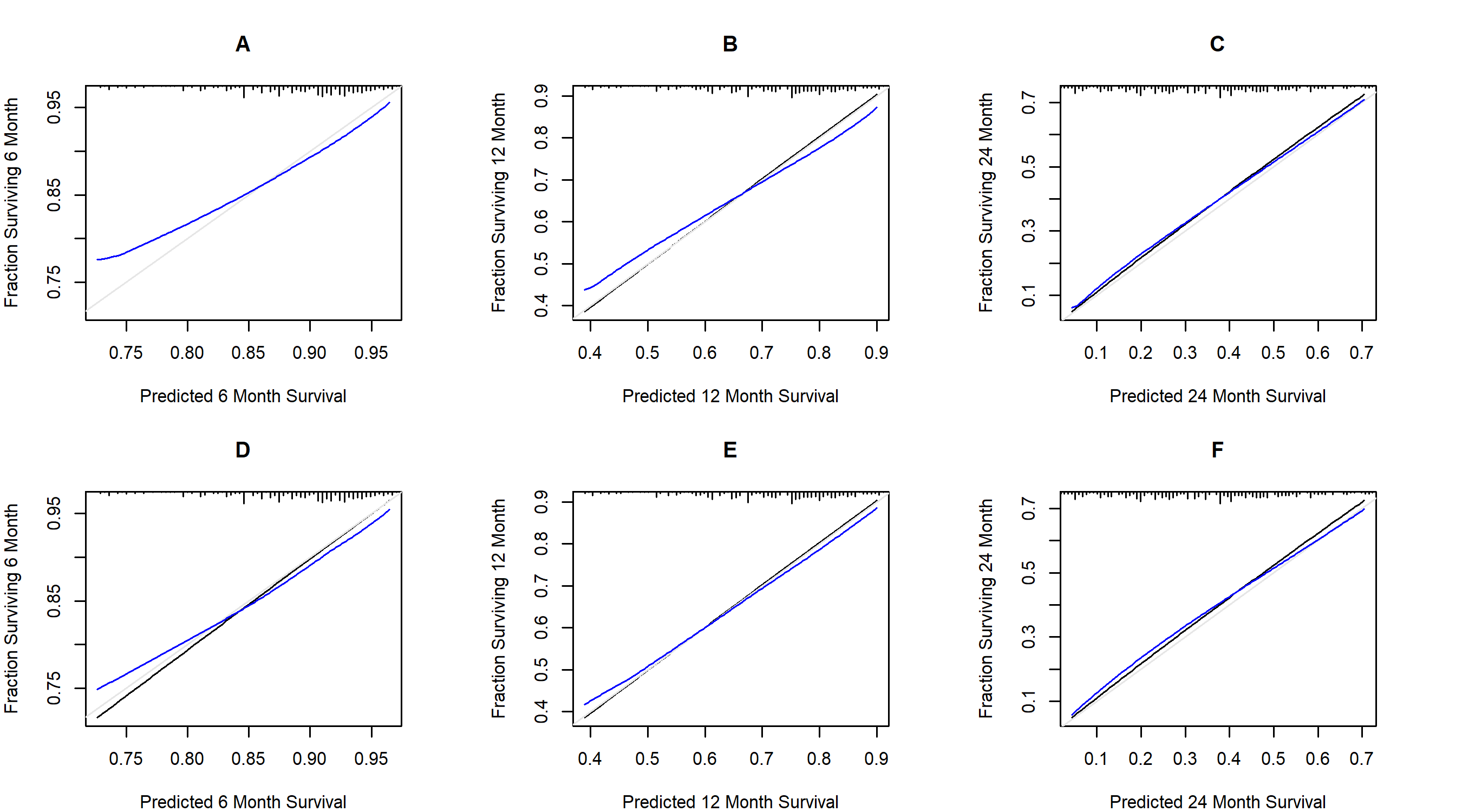


**Supplemental Table 3:** **Final Multivariable Cox Proportional Hazards Results for Overall Survival by Sex using the Validation Dataset (0825)**

|  | **Male Overall Survival** | | | **Female Overall Survival** | | |
| --- | --- | --- | --- | --- | --- | --- |
|  | **HR^1^** | **95% CI^1^** | **p-value** | **HR^1^** | **95% CI^1^** | **p-value** |
| **Age at Diagnosis** | 1.04 | 1.02, 1.05 | <0.001 | 1.03 | 1.01, 1.04 | 0.004 |
| **Karnofsky Performance Status at registration** |  |  |  |  |  |  |
| <=70 | — | — |  | — | — |  |
| 80 | 0.69 | 0.44, 1.09 | 0.109 | 0.99 | 0.61, 1.61 | 0.959 |
| 90 | 0.55 | 0.36, 0.84 | 0.006 | 0.80 | 0.50, 1.29 | 0.357 |
| 100 | 0.49 | 0.30, 0.81 | 0.006 | 0.89 | 0.50, 1.57 | 0.687 |
| **Extent of Resection** |  |  |  |  |  |  |
| GTR | — | — |  | — | — |  |
| STR | 1.19 | 0.91, 1.54 | 0.200 | — | — | — |
| Other | 1.08 | 0.55, 2.14 | 0.824 | — | — | — |
| **MGMT methylation status** |  |  |  |  |  |  |
| Unmethylated | — | — |  | — | — |  |
| Methylated | 0.40 | 0.29, 0.55 | <0.001 | 0.42 | 0.27, 0.63 | <0.001 |
| **Use of Corticosteroids*** |  |  |  |  |  |  |
| No | — | — |  | — | — | — |
| Yes | 1.09 | 0.83, 1.45 | 0.531 | — | — | — |
| **Location of Tumor In Brain** |  |  |  |  |  |  |
| Multiple Sites | — | — |  | — | — |  |
| Frontal Lobe | 0.92 | 0.65, 1.29 | 0.625 | 1.14 | 0.74, 1.75 | 0.559 |
| Occipital Lobe | 0.83 | 0.26, 2.66 | 0.748 | 0.80 | 0.29, 2.24 | 0.670 |
| Parietal Lobe | 0.73 | 0.48, 1.11 | 0.141 | 1.16 | 0.65, 2.04 | 0.617 |
| Temporal Lobe | 0.84 | 0.61, 1.16 | 0.301 | 0.72 | 0.46, 1.14 | 0.160 |
| ^1^HR = Hazard Ratio, CI = Confidence Interval  Variables not included in the table were not included in the final model. | | | | | |  |

**Supplemental Table 4: Final** **Multivariable Cox Proportional Hazards Results for Progression-Free Survival by Sex on Training Dataset (0525)**

|  | **Male Overall Survival** | | | **Female Overall Survival** | | |
| --- | --- | --- | --- | --- | --- | --- |
|  | **HR^1^** | **95% CI^1^** | **p-value** | **HR^1^** | **95% CI^1^** | **p-value** |
| **Age at Diagnosis** | 1.02 | 1.01, 1.02 | <0.001 | 1.03 | 1.02, 1.04 | <0.001 |
| **Karnofsky Performance Status at registration** |  |  |  |  |  |  |
| <=70 | — | — |  | — | — |  |
| 80 | 0.91 | 0.63, 1.32 | 0.614 | 0.78 | 0.53, 1.15 | 0.213 |
| 90 | 0.73 | 0.51, 1.03 | 0.070 | 0.64 | 0.47, 0.88 | 0.006 |
| 100 | 0.73 | 0.51, 1.06 | 0.099 | 0.72 | 0.49, 1.05 | 0.087 |
| **Extent of Resection** |  |  |  |  |  |  |
| GTR | — | — |  | — | — |  |
| STR | 1.16 | 0.94, 1.43 | 0.156 | 1.25 | 0.97, 1.60 | 0.079 |
| Other | 1.79 | 1.06, 3.04 | 0.031 | 1.71 | 0.81, 3.59 | 0.160 |
| **MGMT methylation status** |  |  |  |  |  |  |
| Unmethylated | — | — |  | — | — |  |
| Methylated | 0.49 | 0.38, 0.62 | <0.001 | 0.64 | 0.50, 0.82 | <0.001 |
| **Use of Corticosteroids** |  |  |  |  |  |  |
| No | — | — | — | — | — |  |
| Yes | — | — | — | 1.25 | 0.93, 1.68 | 0.135 |
| **Laterality** |  |  |  |  |  |  |
| Bilateral | — | — |  | — | — |  |
| Left | — | — | — | 1.26 | 0.39, 4.14 | 0.700 |
| Right | — | — | — | 1.80 | 0.55, 5.89 | 0.334 |
| ^1^HR = Hazard Ratio, CI = Confidence Interval | | | | | | |

**Supplemental Figure 3. Male Progression Free Survival (A-F) Calibration curves for the nomogram training data (0525) at 6 months (A), 12 months (B), and 24 months (C) and for independent validation data (0825) at 6 months (D), 12 months (E), and 24 months (F).**


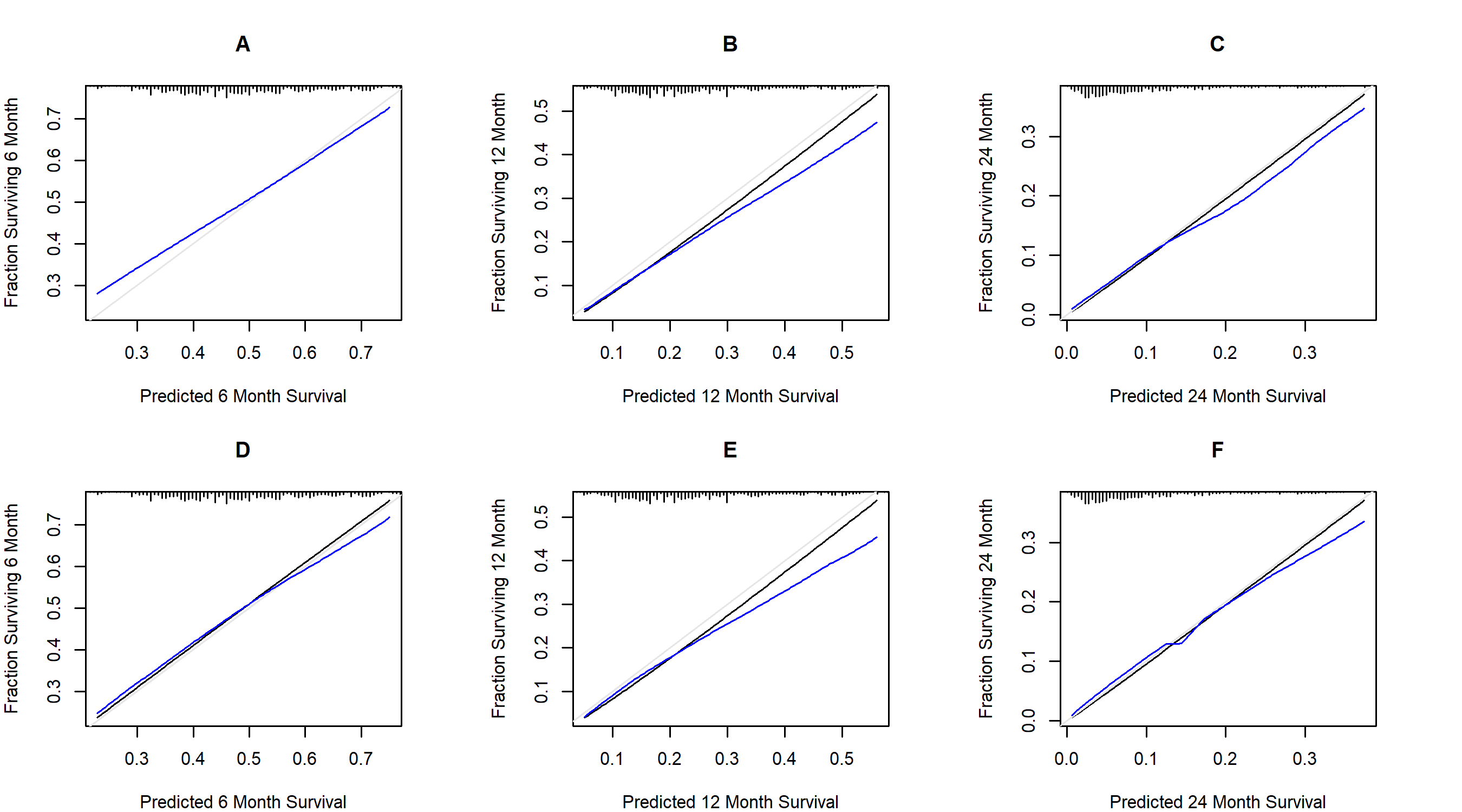


**Supplemental Figure 4. Female Progression Free Survival (A-F) Calibration curves for the nomogram training data (0525) at 6 months (A), 12 months (B), and 24 months (C) and for independent validation data (0825) at 6 months (D), 12 months (E), and 24 months (F).**


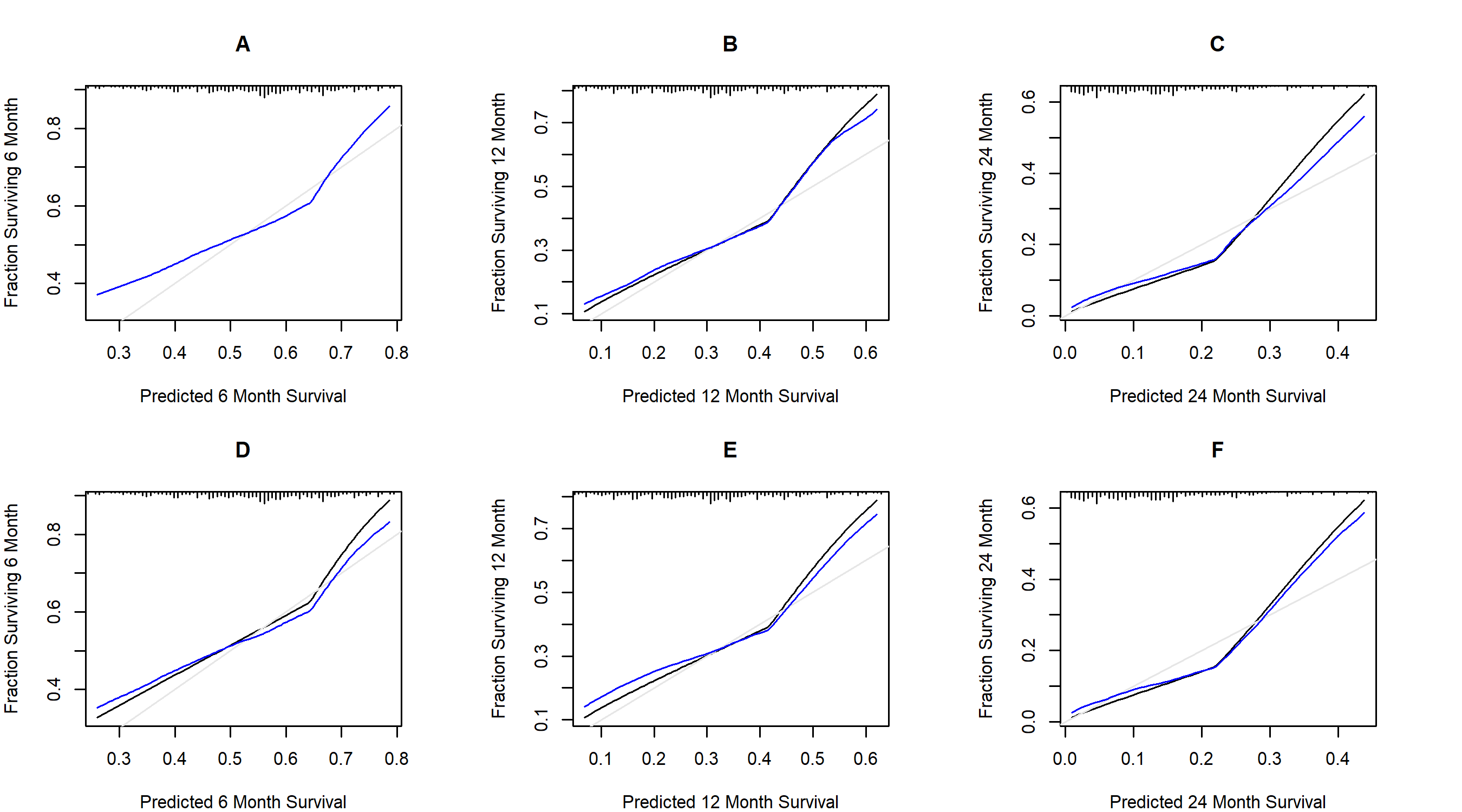

Supplement: Supplementary file 1 — Supplementary file1 (DOCX 296 kb) [file 11060_2021_3886_MOESM1_ESM.docx]
